# The management of large perforations of duodenal ulcers

**DOI:** 10.1186/1471-2482-5-15

**Published:** 2005-06-25

**Authors:** Sanjay Gupta, Robin Kaushik, Rajeev Sharma, Ashok Attri

**Affiliations:** 1 Department of Surgery, Government Medical College and Hospital, Sector 32, Chandigarh 160 030 India

## Abstract

**Background:**

Duodenal ulcer perforations are a common surgical emergency, but literature is silent on the exact definition, incidence, management and complications of large perforations of duodenal ulcers.

**Methods:**

The case files of 162 patients who underwent emergency laparotomy for duodenal ulcer perforations over a period of three years (2001 – 2003) were retrospectively reviewed and sorted into groups based on the size of the perforations – one group was defined as '*small *'perforations (less than 1 cm in diameter), another '*large*' (when the perforation was more than 1 cm but less than 3 cms), and the third, '*giant*'(when the perforation exceeded 3 cm). These groups of patients were then compared with each other in regard to the patient particulars, duration of symptoms, surgery performed and the outcome.

**Results:**

A total of 40 patients were identified to have duodenal ulcer perforations more than 1 cm in size, thus accounting for nearly 25 % of all duodenal ulcer perforations operated during this period. These patients had a significantly higher incidence of leak, morbidity and mortality when compared to those with smaller perforations.

**Conclusion:**

There are three distinct types of perforations of duodenal ulcers that are encountered in clinical practice. The first, are the 'small' perforations that are easy to manage and have low morbidity and mortality. The second are the 'large' perforations, that are also not uncommon, and omental patch closure gives the best results even in this subset of patients. The word 'giant' should be reserved for perforations that exceed 3 cms in diameter, and these are extremely uncommon.

## Background

Duodenal ulcer perforations are a common cause of peritonitis. The classic, pedicled omental patch that is performed for the 'plugging' of these perforations was first described by Cellan-Jones in 1929 [1], although it is commonly, and wrongly attributed to Graham, who described the use of a free graft of the omentum to repair the perforation in 1937 [2]. In this, a strand of omentum is drawn over the perforation and held in place by full thickness sutures placed on either side of the perforation, and this procedure has become the "gold standard" for the treatment of such perforations. However, occasionally, large perforations of the duodenum may be encountered in which there exists the threat of post-operative leakage following closure by this simple method [3,4]. Here, other surgical options such as partial gastrectomy, jejunal serosal patch, jejunal pedicled graft, free omental plug, suturing of the omentum to the nasogastric tube, proximal gastrojejunostomy, or, even, gastric disconnection may be deemed necessary for adequate closure [3-8].

Very little data is available in literature regarding the definition, incidence, and the management of large perforations of duodenal ulcers. This paper represents our experience with the management of this subset of duodenal ulcer perforations over a period of three years from January 2001 to December 2003.

## Methods

A total of 162 patients underwent emergency surgery for duodenal ulcer perforations at our hospital over a period of three years (January 2001 to December 2003). The case files of all these patients were analyzed, and the patients were sorted into four groups according to the size of the perforation noted intra-operatively – Group 1 (less than1 cm perforation); Group 2 (1 cm to 2 cm); Group 3 (2 cms to 3 cms); and, Group 4 (more than 3 cms perforation). No cases of anterior and posterior ulcers, or multiple perforations were encountered while reviewing the operative notes. The technique of omentopexy was essentially the same in all the cases – a total of three sutures were placed onto the normal, healthy duodenum on either side of the perforation, a strand of omentum was placed directly onto the perforation, and the sutures were knotted above this. No attempt was made to close the perforation prior to placing the omentum as a graft.

The case files of all the patients were retrospectively analyzed for patient particulars, intra-operative findings, surgery performed, post-operative stay, morbidity and mortality. The groups were then compared with each other in terms of age, leak rates, hospital stay, morbidity, mortality and the surgery performed. Statistical analysis was done using the *chi-square *and the *t- test *by an independent comparison of each group singly against another by a statistician who was blinded to the study. A *p *value of < 0.05 was taken as significant.

It was found that the perforations between 1 cm and 3 cm in size (Groups 2 and 3 as above) behaved in a similar manner statistically, and therefore, the patients these two groups were combined to give a single group. We finally ended up with 3 groups of perforations – Group A (less than1 cm perforations), Group B (perforations between 1 cm and 3 cms in size), and Group C (more than 3 cms perforation).

## Results

Of the total of 162 patients that underwent emergency surgery for duodenal ulcer perforations at our hospital over three years, there were 148 males (91.36 %) and 14 female (8.64 %) patients, giving a male to female ratio of 10.57 : 1. The average age of the patients was 40.63 years (range 15 – 82 years), with an almost equal age of occurrence for males (40.52 years) and females (41.78 years).

All the patients were divided into three groups as explained above. Group A was deemed to be the small perforation group, Group B was called 'large' perforations, and Group C, 'giant' perforations. The majority of patients came under the 'small' perforation group, but there were 38 patients (23.46 %) with large perforations as per our definition. These patients had a higher age of presentation (47.18 years) than the patients with smaller perforations (39.46 years). Giant perforations, or perforations greater than 3 cms in size were seen only 2 cases, accounting for a small percentage (1.23 %) of all cases seen.

When the small perforation group was compared with the larger perforations, it was found that the large perforations had a higher morbidity (*x*^*2 *^= 37.4503, *p *< 0.05), leak rate (*x*^*2 *^= 4.9117, *p *< 0.05), and hospital stay (*t value *5.117, *p *< 0.001) and that this difference was statistically significant. This therefore, lends support to the popular opinion that large perforations have a worse outcome.

Overall, the commonest surgery performed was the Cellan-Jones omental patching – in 119 of the 122 cases in Group A; 30 of the 38 patients in Group B. When the results of omental patch were compared between the two groups, no significant difference was found in the leak rates (*x*^*2 *^= 2.8698; *p *> 0.5) and mortality (*x*^*2 *^= 1.4732; *p *> 0.1), thereby implying that this was an equally effective method for the closure of larger perforations also. Jejunal serosal patch using a loop of the jejunum, and antrectomy (4 cases each) were the other surgeries performed in Group B, when closure with the omentum was thought to be unsafe by the operating surgeon. Five (12.5 %) patients of this group had a leak following closure of the perforation; 3 following omental patch and 1 each after performance of jejunal serosal patch and antrectomy, whereas only 3 cases developed leak in Group A (2 after omental patch and 1 after truncal vagotomy and pyloroplasty).

Two cases had 'giant' perforations extending onto the pylorus – in one, resection and Billroth II reconstruction was performed, and in the other, jejunal serosal patch. The patient who underwent resection had presented late, and succumbed to septicaemia on the very first post-operative day. The other remained well and was discharged on the 11^th ^post-operative day.

Overall, the patients with large perforations (Group B) had significantly increased hospital stay, leak rates, and morbidity (Table 1). The hospital stay was almost double for these patients (13.65 days versus 6.93 days). Although the overall morbidity was 48.76 %, it was much higher in the larger perforations (groups B and C). The common morbidity encountered was chest infections (39 cases), but wound infection (12 cases), biliary leak (08 cases), intra-abdominal abscesses (06 cases), burst abdomen (06 cases), renal failure (02 cases), DIC (04 cases), jaundice and upper gastrointestinal bleeding (01 case each) were also recorded. The mortality in this series was 8.64 % (14 cases), and again, it was significantly higher in perforations more than 1 cm in size (*x*^*2 *^= 3.8940; *p *< 0.05). Table 1 gives the details of all the three groups.

**Table 1:** Patient data

	**Group A – 'Small' (Less than 1 cm)**	**Group B – 'Large' (1 cm – 3 cm)**	**Group C – 'Giant' (More than 3 cm)**
**Number of cases**	122 (75.31 %)	38 (23.46 %)	02 (1.23 %)
**Average age**	39.46 years	47.18 years	37.50 years
**Male/Female**	109 : 13	37 : 1	2 : 0
**Average Duration of Symptoms**	2.5 days	3.18 days	3.50 days
**Surgery Performed**	Omental Patch 119 ** Pyloroplasty 03 *	Omental Patch 30 *** Jejunal Serosal Patch 04 * Antrectomy 04 *	Antrectomy and Billroth II 01 Jejunal Serosal Patch 01
**Post-operative Leak**	03 (2.46 %)	05 (13.16 %)	-
**Morbidity**	41	37	01
**Post-operative Hospital Stay**	6.93 days	13.65 days	6.00 days
**Mortality**	07 (5.74 %)	06 (15.79 %)	01 (50 %)

Each * indicates one post-operative leak

## Discussion

Duodenal ulcer perforation is a common surgical emergency in our part of the world. The overall reported mortality rate varies between 1.3 to nearly 20 % [9-11] in different series, and recent studies have shown it to be around 10 % [11]. Factors such as advancing age, concomitant disease, preoperative shock, size of the perforation, delay in presentation and operation, have all been defined by various authors to be risk factors for mortality in such a situation [9-11]. Although the size of a perforation is an important measure in determining the outcome, a review of literature failed to reveal, any accepted definition of either small or giant perforations of duodenal ulcers. Neither could we come across any specific recommendations regarding the management of giant / large perforations, which are said to be "difficult" to manage and have anecdotally been associated with high leak rates and mortality. This is in contrast to the well accepted and documented definition of giant duodenal ulcers (more than 2 cms in size), which may or may not perforate, but are usually considered to be an indication for definitive, elective ulcer surgery [8,12].

Commonly, duodenal ulcer perforations are less than 1 cm in greatest diameter, and as such, are amenable to closure by omentopexy [3]. Our experience does seem to validate this, and this subset of 'small' perforations does seem to have the best outcome. It is the perforations that are larger that have been the cause of much confusion in their definition and management. The size of such 'giant' sized perforations has arbitrarily been defined by various authors as being greater than 0.5 cms [7], 1 cm [3,4], or 2.5 cms [6] in greatest diameter, but we failed to uncover any specific size in available English language literature beyond which to label these perforations as "giant". These perforations are considered particularly hazardous because of the extensive duodenal tissue loss and surrounding tissue inflammation, which are said to preclude simple closure using omental patch, often resulting into post-operative leak or gastric outlet obstruction [3,4]. The tendency to leak may further be aggravated by the high intraluminal pressures, extrusion of the duodenal mucosa through the closure, and, autodigestion by the pancreatic enzymes and bile, thereby further compromising an already sick patient [13].

Our data seems to suggest that based on the size, duodenal perforations can be classified into three main groups (1) *small *perforations that are less than 1 cm in size, and have the best outcome; (2) *large *perforations, that have a size between 1 cm and 3 cms; and, (3) *giant *perforations that exceed 3 cm size. The usage of the word 'giant' for a duodenal perforation should be restricted to such large defects, where omentopexy may be deemed unsafe, and other options may be thought to be necessary.

In the absence of any specific definition and guidelines regarding the management of such large / giant perforations in literature, different authors have recommended varied surgical options from time to time, based on their experience and research. These have included resection of the perforation bearing duodenum and the gastric antrum in the form of a partial gastrectomy, with reconstruction as either a Billroth I or II anastomosis, or the more morbid procedure of gastric disconnection in which vagectomy, antrectomy, gastrostomy, lateral duodenostomy and feeding jejunostomy are performed, with restoration of intestinal continuity electively after 4 weeks of discharge [8]. Others have recommended conversion of the perforation into a pyloroplasty, or, closure of the perforation using a serosal patch or a pedicled graft of the jejunum, or, the use of a free omental plug to patch the defect, and even, suturing of the omentum to the nasogastric tube [3-8]. Proximal gastrojejunostomy and / or vagotomy may be added to these procedures to provide diversion and a definitive acid reducing procedure respectively [8]. However, as can be appreciated, each of these procedures not only prolongs the operating time, but also requires a level of surgical expertise that may not be available in the emergency [6]. In addition, each of these procedures has it own morbidity that may add up significantly to alter the final outcome of the patient, and more importantly, none of them is immune to the risk of leak in the post-operative period, which has been the main concern against performing the omental patch in larger perforations [3,4].

The results of omentopexy in small and large sized perforations in the present series give statistically similar results. The leak rates and mortality of the two groups after omentopexy remain comparable, thereby suggesting that this may be considered as the procedure of choice in all perforations upto a size of 3 cms. The procedure is simple and easy to master, and, avoids the performance of a major resection in a patient who is already compromised. In fact, Sharma et al also reported the success of the omental plug in perforations of duodenal ulcers more than 2.5 cms in size; only, they preferred using a free graft of the omentum rather than a pedicled one [6]. We feel that mobilization of the omentum on its pedicle from the colon, and placement of sutures into the normal duodenum away from the perforation makes the performance of omental patch safe even in the presence of large sized perforations.

In the present series, only 2 cases were defined to be 'giant' according to the size (more than 3 cm) that we have defined – one underwent antrectomy and Billroth II reconstruction, the other, a jejunal serosal patch. The first patient (antrectomy) succumbed to the ongoing septicaemia on the very first post-operative day, but the other patient survived. This is the group of patients with truly giant perforations who need to be analyzed further to determine the best course of action i.e. resectional versus non-resectional surgery. However, the less number of patients in this group did not allow us to reach any definite conclusion regarding their ideal management. Further study is needed to optimize our efforts to this target group.

## Conclusion

Duodenal perforations should be classified as small, large or giant according to their size encountered at laparotomy. In the emergency setting, such patients are often seriously ill and it is not advisable to perform major surgical procedures on them. The Cellan-Jones omental patch is simple, can be performed in a relatively short time, and remains dependable even for the closure of large sized perforations (i.e. perforations upto 3 cms in size). The addition of a feeding jejunostomy and placement of a tube drain in the Morrison's space may offer a further sense of ''security'' to the operating surgeon, keeping by open the option of maintaining the nutrition of the patient as well as creating a controlled duodenal fistula in case of a post-operative leak. The word ''giant'' should be reserved only for perforations that exceed 3 cm in diameter.

## Competing interests

The author(s) declare that they have no competing interests.

## Authors' contributions

**SG **carried out acquisition, statistical analysis and interpretation of the data and drafting of the initial manuscript

**RK **conceptualized the paper, and was involved in the interpretation of data, drafting of the manuscript, and revising it critically for the intellectual content

**RS **carried out drafting of the manuscript, and revised it critically for the intellectual content till the final version was reached

**AKA **helped in the revisions of the intellectual content and gave final approval of the version to be published.

All authors have read and approved the final manuscript.

## Pre-publication history

The pre-publication history for this paper can be accessed here:


